# Pathological alterations in liver injury following congestive heart failure induced by volume overload in rats

**DOI:** 10.1371/journal.pone.0184161

**Published:** 2017-09-21

**Authors:** Mohammed Shaqura, Doaa M. Mohamed, Noureddin B. Aboryag, Lama Bedewi, Lukas Dehe, Sascha Treskatsch, Mehdi Shakibaei, Michael Schäfer, Shaaban A. Mousa

**Affiliations:** 1 Department of Anaesthesiology and Intensive Care Medicine, Charité University Berlin, Campus Virchow Klinikum and Campus Charité Mitte, Berlin, Germany; 2 Department of Anatomy, Ludwig-Maximilians-University Munich, Munich, Germany; Indian Institute of Toxicology Research, INDIA

## Abstract

Heart failure has emerged as a disease with significant public health implications. Following progression of heart failure, heart and liver dysfunction are frequently combined in hospitalized patients leading to increased morbidity and mortality. Here, we investigated the underlying pathological alterations in liver injury following heart failure. Heart failure was induced using a modified infrarenal aortocaval fistula (ACF) in male Wistar rats. Sham operated and ACF rats were compared for their morphometric and hemodynamic data, for histopathological and ultrastructural changes in the liver as well as differences in the expression of apoptotic factors. ACF-induced heart failure is associated with light microscopic signs of apparent congestion of blood vessels, increased apoptosis and breakdown of hepatocytes and inflammatory cell inifltration were observed. The glycogen content depletion associated with the increased hepatic fibrosis, lipid globule formation was observed in ACF rats. Moreover, cytoplasmic organelles are no longer distinguishable in many ACF hepatocytes with degenerated fragmented rough endoplasmic reticulum, shrunken mitochondria and heavy cytoplasm vacuolization. ACF is associated with the upregulation of the hepatic TUNEL-positive cells and proapoptotic factor Bax protein concomitant with the mitochondrial leakage of cytochrome C into the cell cytoplasm and the transfer of activated caspase 3 from the cytoplasm into the nucleus indicating intrinsic apoptotic events. Taken together, the results demonstrate that ACF-induced congestive heart failure causes liver injury which results in hepatocellular apoptotic cell death mediated by the intrinsic pathway of mitochondrial cytochrome C leakage and subsequent transfer of activated caspase 3 into to the nucleus to initiate overt DNA fragmentation and cell death.

## Introduction

Chronic heart failure, a progressive disease marked by repeated hospitalizations for episodes of acute decompensation, is frequently complicated by liver dysfunction—one of the most important risk factors for poor clinical outcome and death [1, 2]. Particularly right heart failure causes liver congestion which over time leads to liver stiffness and finally may result into liver fibrosis [3, 4]. This can induce a constellation of histopathological changes that can range from mild sinusoidal dilation to advanced fibrosis [3, 4]. In addition, the heart may not be able to deliver oxygen at a rate proportionate to the demands of the metabolizing tissues resulting in damage to other organ systems such as liver, although this is subject of debate [5, 6]. The liver is particularly sensitive to oxygen lack and the integrity of the liver cell depends on an adequate oxygen supply [7, 8]. Interestingly, in an animal model of caval vein clamping it was shown that liver congestion triggers liver stiffness with the end result of liver fibrosis without an overt inflammatory process [9]. There are only very few reports of histopathological alterations in liver sections obtained by aspiration biopsy or necropsy from patients with congestive heart failure [3].

Recently, a small clinical study investigated the predominant hepatic cell death pattern in heart failure by determining serum levels of hepatic cytokeratin-18 epitopes (M65 and M30) which constitute peripheral cell death markers [1]. These elevated serum markers gave first indirect evidence that apoptotic cell death seems to contribute to liver injury following heart failure. It is well established that apoptotic signals initiate specific cellular pathways such as the disruption of mitochondrial transmembrane potential, followed by the release of mitochondrial proteins like cytochrome C. The subsequent activation of caspase subtypes within the apoptosome complex finally leads to cell death [10–12]. Once cytochrome C is solubilized, permeabilization of the outer mitochondrial membrane by Bax is sufficient to allow the release of this protein from isolated liver cell mitochondria into the extramitochondrial environment [13]. Consistently, previous studies by Seervi [14] substantiated that mitochondrial cytochrome C release was critical and essential for the caspase activation in cell systems.

The primary goal of this study was to investigate in a modified experimental rat model of congestive heart failure the histopathological and ultrastructural changes in the liver. In addition, we examined the expression of apoptotic cell death markers such as caspase 3, activated caspase 3, mitochondrial enzyme cytochrome C and Bax.

## Material and methods

### Animals

Male Wistar rats, 280–300 g (Harlan Winkelmann, Borchen, Germany), were maintained on standard laboratory rat chow and water ad libitum. The animals were kept in cages (n = max. 5) on a 12-h light–dark cycle. This study was carried out in accordance with the European Directive introducing new animal welfare and care guidelines (2010/63/EU). IRB approval for animal experiments was given by local authorities (Reg # G 0144/12; Landesamt für Gesundheit und Soziales, Berlin, Germany) after a thorough review process. All surgical interventions were performed under isoflurane (ACF induction) and tiletamine/zolazepam (hemodynamic measurements) anesthesia and all efforts were made to minimize animals’ suffering.

### Experimental model

The needle-technique to induce an infrarenal aortocaval fistula (ACF) has previously been described by Garcia and Diebold using a 18G needle [15, 16]. Here, we applied a modified technique using a 16G needle [16]. Briefly, under isoflurane anesthesia a laparotomy was performed and the aorta was punctured with a 16G disposable needle (Braun, Melsungen, Germany) distal to the renal arteries. Then, the needle was advanced to penetrate the aortic wall into the adjacent inferior vena cava. After temporarily compressing the aorta and venous vessels above and below the puncture site, the needle was carefully withdrawn and the aortic puncture site was sealed with a drop of cyanoacrylate glue [16]. Patency of the fistula (ACF) was visualized by the pulsatile flow of oxygenated blood from the aorta into the inferior vena cava [16]. Sham operated animals underwent a laparotomy with a temporary compression of the vessels without any puncture of the aorta. As analgesic regimen, animals received intraoperatively a s.c. injection of 40 mg/kg dipyrone which was continued over the next three postoparative days by dipyrone dissolved in drinking water (1.33mg/ml). The rats were maintained in animal room facilities and monitored daily under the supervision of a veterinarian. Considering technical aspects of the here presented approach, the main complication was uncontrollable bleeding at the puncture site of the ACF induction (mortality: 2 out of 40 rats). During the progressive course of heart failure over 28±2 days 3 out of 40 rats died due to cardiac arrhythmias. Out of the remaining 35 rats n = 5 sham rats and n = 10 ACF rats were used for morphometric measurements, hemodynamic measurements, rBNP plasma concentrations, and Western blot. In addition, n = 5 sham rats and n = 5 ACF rats were used for light microscopy/immunohistochemistry and n = 5 sham rats and n = 5 ACF rats were used for electron microscopy.

### Morphometric data

After 28±2 days of fistula induction animals were sacrificed in isoflurane anesthesia and blood, heart, lung and liver tissue were quickly removed. The wet weight of heart, lung and liver tissue was measured by a weighing balance and normalized to the body weight of the individual animal to obtain the respective indices.

### rBNP plasma concentration

Blood samples were taken from animals into EDTA-preloaded tubes. Then, the blood was centrifuged at 4°C at 1,000g for 10 min and the plasma was maintained at -80°C until extraction. Plasma rBNP45 concentrations were measured by using a sensitive enzyme linked immunosorbent assay (ELISA) kit (Abnova, Heidelberg, Germany) [16].

### Hemodynamic parameters

For hemodynamic measurements of animals, the “closed chest” method in spontaneously breathing rats was used as described previously [16, 17]. All measurements were performed under tiletamine /zolazepam anesthesia (Zoletil^®^, 10 mg/kg s.c. followed by 50 mg/kg i.m.) 28±2 days after fistula induction [16–18]. Measurements were registered and analyzed by the PowerLab^®^-system/-software (AD Instruments, New Zealand). After tracheotomy, a PE-50 tubing catheter was inserted via the left jugular vein into the superior vena cava for assessment of central venous pressure. Arterial blood pressure was measured by cannulating the right carotid artery with a micro-tip pressure-volume conductance catheter (Millar®, SPR- 838 NR). Intra-ventricular pressures and volumes were registered by further advancing the catheter into the left ventricle and optimizing its position aiming for maximal stroke volume (SV). For measurement of the parallel conductance volume 100 μl of 15% saline was injected into the central venous line as a correction factor for the blood–left ventricle (LV) tissue interface. Heart rate was derived from the ECG signal. After completion of the hemodynamic measurements animals, which were still under anesthesia, were killed by exsanguination and organs were eviscerated for further determinations.

### Tissue preparation

For histology and immunohistochemical experiments rats were deeply anesthetized with isoflurane and transcardially perfused with 100 ml 0.1 m PBS (pH 7.4) and 300 ml cold PBS containing 4% paraformaldehyde and 0.2% picric acid (pH 7.4; fixative solution) for light microscopic immunohistochemistry and with 4% paraformaldehyde/0.1% glutaraldehyde/0.2% picric acid solution (pH 7.4) for electron microscopy, respectively [19]. The liver tissue was removed, postfixed for 90 min at 4 C in the fixative solution, and cryoprotected overnight at 4 C in PBS containing 10% sucrose. The tissues were then embedded in Tissue-Tek compound (OCT, Miles, Inc., Elkhart, IN) and frozen. Consecutive sections (6 μm thick) prepared by using a cryostat were mounted onto gelatin-coated slides.

### Histological examination

Serial 6 μm thick sections of liver tissues were cut and stained with hematoxylin-eosin as previously shown (Mayer 1981) [20]. All stained sections were examined by two experienced pathologists, blinded for the sample assignment to the different experimental groups. All H&E-stained slices were reviewed for changes in hepatic tissues under light microscopy (Zeiss Axioplan photomicroscope equipped with a digital camera), by two experienced pathologists blinded to the respective treatments. Cellular swelling, deep nuclear staining, nuclear shrinking, karyoclasis, nuclear dissolving, inflammatory cell penetration and hepatic sinus structure were all recorded. Random fields (minimum five areas) from each section (n = 5 rats) were captured using a ×40 magnification lens. Hepatic glycogen content was analyzed by Periodic acid-Schiff staining. However, hepatic collagen content was analyzed by Sirius red staining (saturated picric acid containing 0.1% Sirius Red F3B) of paraffin embedded sections to assess the degree of fibrosis. The Sirius red-positive area was measured in four individual fields on each slide and quantified using NIH imaging software. Also, Oil Red O stain highlighting fat globules in a frozen section of the liver was performed. Briefly, frozen liver sections incubated with Oil Red O in 60% isopropanol for 20 minutes. The sections were then washed with 60% isopropanol for 30 seconds, treated with hematoxylin for 2 minutes, and washed in cold water. The coverslips were mounted on microscope slides.

### Electron microscopy

Liver tissue was processed for electron microscopy as described previously [19–21]. Small tissue pieces were postfixed in 1% tannic acid (in 0.1 m phosphate buffer) and 1% osmium tetroxide solution (in 0.1 m PBS), dehydrated in ethanol, and embedded in Epon. Semithin and ultrathin sections were cut on a Reichert Ultracut (Leica, Nussloch, Germany), and the ultrathin sections were contrasted with 2% uranyl acetate/lead citrate. Finally, the ultrathin sections were examined under a transmission electron microscope (TEM 10, Zeiss, Jana, Germany). Semithin sections of samples were stained 1 to 2 minutes in 1% Toluidine Blue (Merck, Darmstadt, Germany), rinsed several times in purified water, and examined under a light microscope (Axiophot 100; Zeiss, Jena, Germany).

### Apoptosis assay

The staining method for liver apoptosis was performed in situ using Chemicon Apo-Direct Tunel Assay Kit (Merck Millipore, Darmstadt, Germany) for the detection of the internucleosomal DNA fragmentation, characteristic of apoptosis according to the manufacturer’s instructions. Briefly, 6 μm sections of paraformaldehyde-fixed, liver tissue were postfixed with 4% formaldehyde/PBS for 30 min at 4°C, permeabilized with proteinase K at room temperature for 15 min and 0.2% Triton X-100/PBS for 15 min at 4°C, and incubated with a mixture of nucleotides and TdT enzyme for 60 min at 37°C. The reaction was terminated with 2 SSC, and the sections were washed with PBS. After that, staining was completed by a 1 min incubation with 4`6-diamidino-2-phenylindole dihydrochloride (DAPI; Sigma), and coverslips were mounted on slides. Fluorescence nuclei were detected by visualization with a microscope equipped with fluorescein filters (IX70; Olympus, Melville, NY). As a negative control, sections were incubated in the absence of TdT enzyme. The degree of apoptosis was estimated by counting the number of TUNEL positive apoptotic cells were counted in fifteen random fields in each slide from liver tissues at a microscopic magnification of x400. Only cells that displayed DAPI and intensely fluorescence nuclei by TUNEL assay were counted as apoptotic. The data were expressed as the mean number of TUNEL-positive cells/field.

### Double immunofluorescence staining

Double immunofluorescence staining of cytochrome C in the liver was performed as described previously [22, 23]. Liver sections were incubated overnight with the following primary antibodies: 1) monoclonal mouse anti-mitochondrial antibody (clone # MTC02; # cat MA5-12017, Thermo Fisher Scientific Inc, Rockford, IL, USA) in combination with rabbit polyclonal anti-cytochrome C (clone # c 136 F3, cat #4280, Cell Signalling, Danvers, MA, USA); 2) mouse monoclonal anti-rat CD45 (Leukocyte common antigen, LCA, clone # OX-1, cat #202201, AbD Serotec, Puchheim, Germany), rabbit polyclonal anti-cleaved caspase 3 (clone # Asp175, cat # 9661, Cell Signalling, Danvers, MA, USA), rabbit polyclonal anti-caspase 3 (cat #ab13847, Abcam; Cambridge, MA) or rabbit polyclonal anti-Bax (clone # P-19, cat # SC-526, Santa Cruz biotechnology, Inc, Texas, USA). After incubation with primary antibodies, the tissue sections were washed with PBS and then incubated with red fluorescent Alexa Fluor 594 donkey anti-rabbit antibody (Vector Laboratories) in combination with green fluorescent Alexa Fluor 488 goat anti-mouse (Invitrogen, Germany). Thereafter, sections were washed with PBS, and the nuclei stained bright blue with 4'-6-Diamidino-2-phenylindole (DAPI) (0.1 μg/ml in PBS) (Sigma). To demonstrate specificity of the staining, the following controls were included by omission of either the primary antisera or the secondary antibodies.

Finally, the tissue sections were washed in PBS, mounted on vectashield (Vector Laboratories) and imaged on a confocal laser scanning microscope, LSM510, equipped with an argon laser (458/488/514 nm), a green helium/neon laser (543 nm), and a red helium/neon laser (633 nm; Carl Zeiss, Göttingen, Germany). Single optical slice images were taken using x10 or x20 Plan-Neofluar air interface or x40 Plan-Neofluar oil interface objective lens.

### Quantification of immunostaining

Quantification of immunofluorescent colocalization of mitochondrial marker with cytochrome C in liver tissue sections was performed by using the Zeiss Zen 2009 software Carl Zeiss Micro-Imaging GmbH (Göttingen, Germany). Colocalization of proteins of interest was quantified by calculating the overlap and colocalization coefficient as derived from Mander’s article based on Pearson’s correlation coefficient [24]. The settings of the confocal microscope were established using a control section and kept unchanged for all subsequent acquisitions. Images were thresholded to exclude background fluorescence and gated to include intensity measurements only from positively stained cells. Images were adjusted to a threshold to exclude background fluorescence and gated to include intensity measurements only from positively stained cells. Six to eight images were sampled per animal using 40x objective lens. The number of caspase 3 stained nuclei was determined by the formula: caspase-3 stained nuclei/total number of DAPI stained nuclei ×100. For images analysis, using area of the whole stained tissue section (μm^2^).

### Western blot analysis

Liver tissue from rats of the different experimental groups were removed from animals and solubilized according to Mousa et al. [19, 22] to obtain total cell protein. Then the Western blot analysis was performed as previously described [19, 22]. Western blot bands of cleaved caspase-3 were quantified by Java Image processing and analysis software (ImageJ, Version 1.38x; open-source image software) as described previously [19, 22].

### Statistical analysis

When data were analyzed, they were represented as means ± SD. Normal distribution was analyzed with the Kolmogorov-Smirnov test. Sample comparisons between the ACF- and Sham-group were made using two-sided Student t-test in the case of normally distributed data and Mann-Whitney-U test in the case of data not distributed normally. Differences were considered significant if *P<0*.*05*. All tests were performed using Sigma Plot 13.0 statistical software.

## Results

### Increased heart and lung weight indices and rBNP-45 plasma concentrations following congestive heart failure

**Fig 1:**
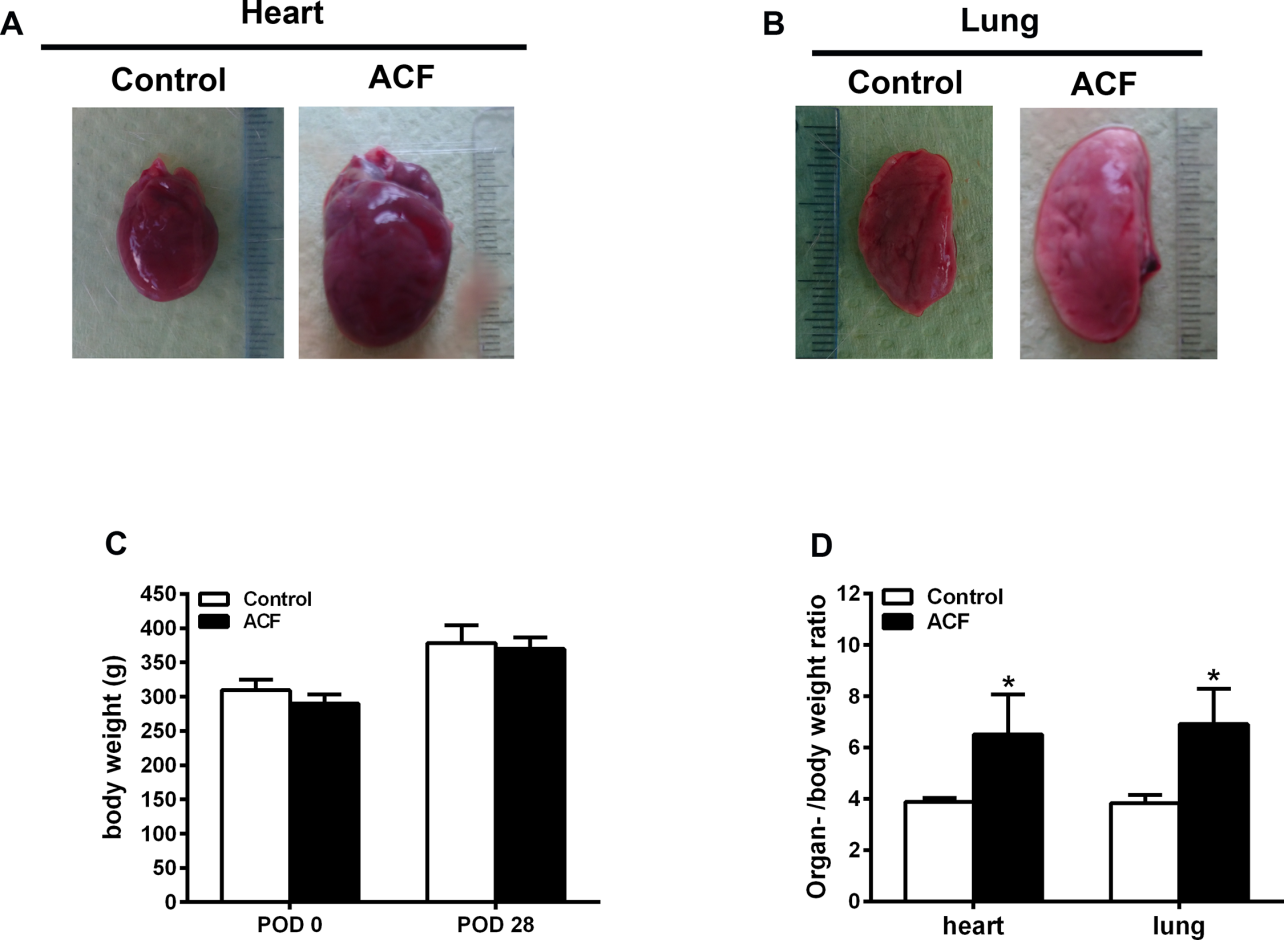
Heart and lung morphological changes following ACF-induced congestive heart failure. Increased heart and lung weight indices (**A, B, D**) but not body weight (**C**) following ACF-induced congestive heart failure. Note, that heart and lung weight indices were significantly increased at 28 days of ACF rats (n = 10) compared to those of sham operated controls (n = 5) (heart index: ACF 6.5±1.6 versus Controls 3.9±0.2; lung index: ACF 6.9±1.4 versus Controls 3.8±0.3; p<0.01, Student t-test) (**D**). Data show means ± SD.

Heart and lung weights as well as heart and lung indices (related to body weight) were significantly increased at 28±2 days after ACF-induced heart failure compared to sham operated controls (p<0.05, Student t-test) (Fig 1A, 1B and 1D), whereas body weights were not significantly different between both groups (Fig 1C). Values for rBNP-45 plasma concentrations were significantly increased in ACF rats (147.0±5.9 pg/ml) compared to controls (28.1±3.5 pg/ml) (p<0.05, Student t-test).

### Systolic and diastolic dysfunction in ACF rats

*In vivo* hemodynamic measurements showed that central venous (CVP) (p<0.05, Student t-test; controls: 0.5±0.3 mmHg; ACF: 5.5±0.4 mmHg) and left end-diastolic pressure (LVEDP) were significantly increased in ACF rats (p<0.01, Student t-test; controls: 5.1±0.3 mmHg; ACF: 12.1±1.0 mmHg). While the left ventricular end diastolic (LVEDV, ACF: 636±139 μl versus Controls: 194±22.4 μl) and end systolic volumes (LVESV, ACF: 404±101 versus Controls: 53±14 μl) were significantly elevated (p<0.05, Mann Whitney U test), the left ventricular ejection fraction (LVEF) (p<0.05, Student t-test; controls: 74±2.0%; ACF: 45±4.0%) and the maximum rate of pressure development (dP/dt max)(p<0.05, Student t-test; controls: 15940±2143 mmHg/s; ACF: 8851.3±3270 mmHg/s) were significantly reduced.

### Histopathological alterations in the liver following congestive heart failure

**Fig 2:**
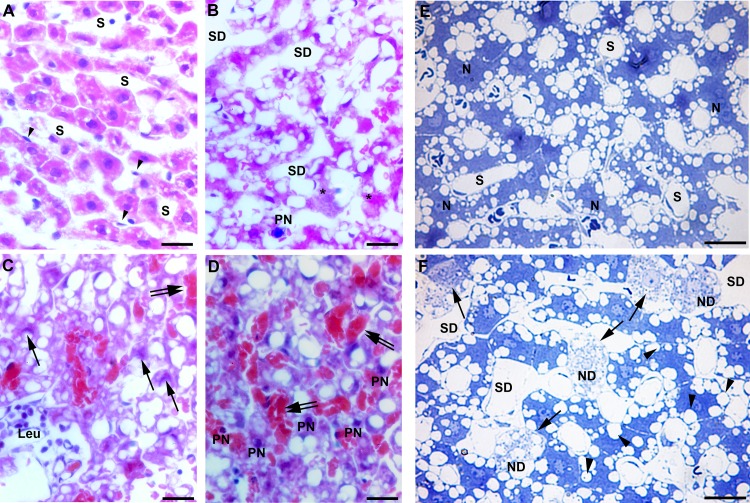
Light microscopic photographs of representative haematoxylin-eosin and toluidine blue stained liver sections. **A**) Note normal lobular structure with hepatocytes having prominent rounded nuclei. **B-D**) Liver sections of ACF rats show pyknotic nuclei (PN), cytoplasmic vacuolization, sinusoidal dilation (SD), leukocyte infiltration (Leu) massive breakdown of hepatocytes (*), and congestive blood vessels (double arrows) as well as a crescent-shaped condensation of nuclear chromatin (arrow). **E**) Semithin liver sections of control rats were stained in 1% toluidine blue showing normal branching and anastomosing hepatocyte cords separated by hepatic blood sinusoids. Note, hepatocytes contain prominent rounded nuclei (N). **F**) Semithin liver sections of ACF rats show hepatocytes with large vacuoles (arrow head) in the periportal area, heterogeneous parenchyma, which consists of dark and compact hepatocytes flanked by ballooned cells (also called apoptotic cells) (arrow) with nuclear degeneration (ND). Bars = 20 μm.

**Fig 3:**
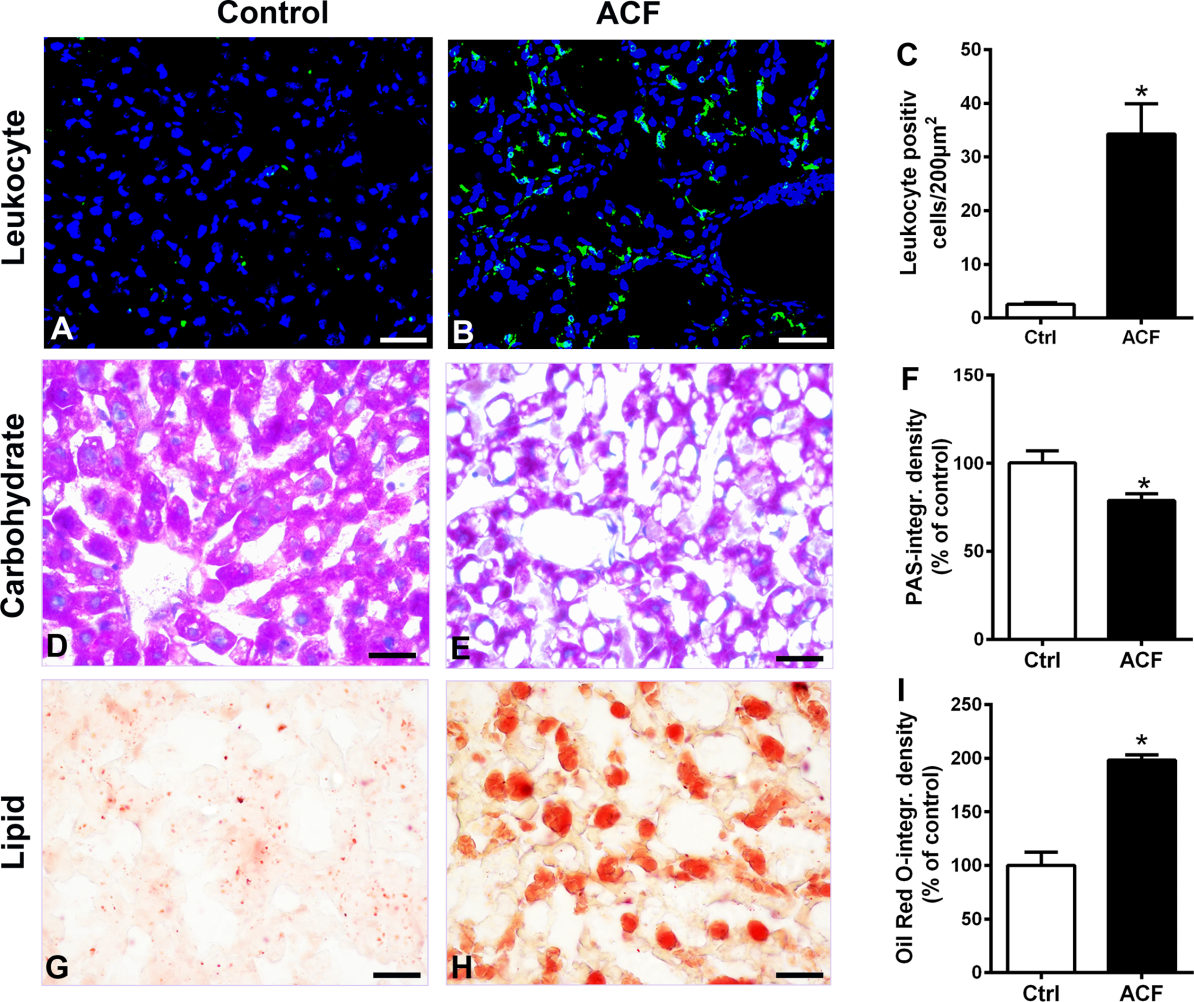
Light microscopic photographs of representative pan-leukocyte marker CD45 immunohistochemistry as well as Periodic acid-Schiff (PAS) and Oil Red O staining of liver sections. **A-C**) show leukocyte infiltrations as detected with pan-leukocyte marker CD45 (green fluorescence) with DAPI-counterstained nuclei (blue fluorescence) within the liver sinusoids. Quantification of CD45^+^ leukocyte positive cells/200 mm^2^ showed significantly more leukocytes in ACF rats (34.3±5.6) compared to controls (2.5±0.3)(p<0.05, Student t-test). **D-F**) show that the glycogen granules of the hepatic cells as examined in PAS-stained sections of liver showed a significant decrease in glycogen granules in ACF rats (78.8±3.8) compared to control (100±2.8)(p<0.05, Student t-test). **G-I**) Representative Oil Red O stain highlighting fat globules in a frozen section of the liver revealed the presence of large (macrovesicular) fat globules only in ACF rats (n = 5) compared to controls (n = 5)(PAS-optical density ACF: 198.3±4.9% versus controls: 100±12.4%) p<0.05, Student t-test). Bars = 20 μm. Data show means ± SD.

**Fig 4:**
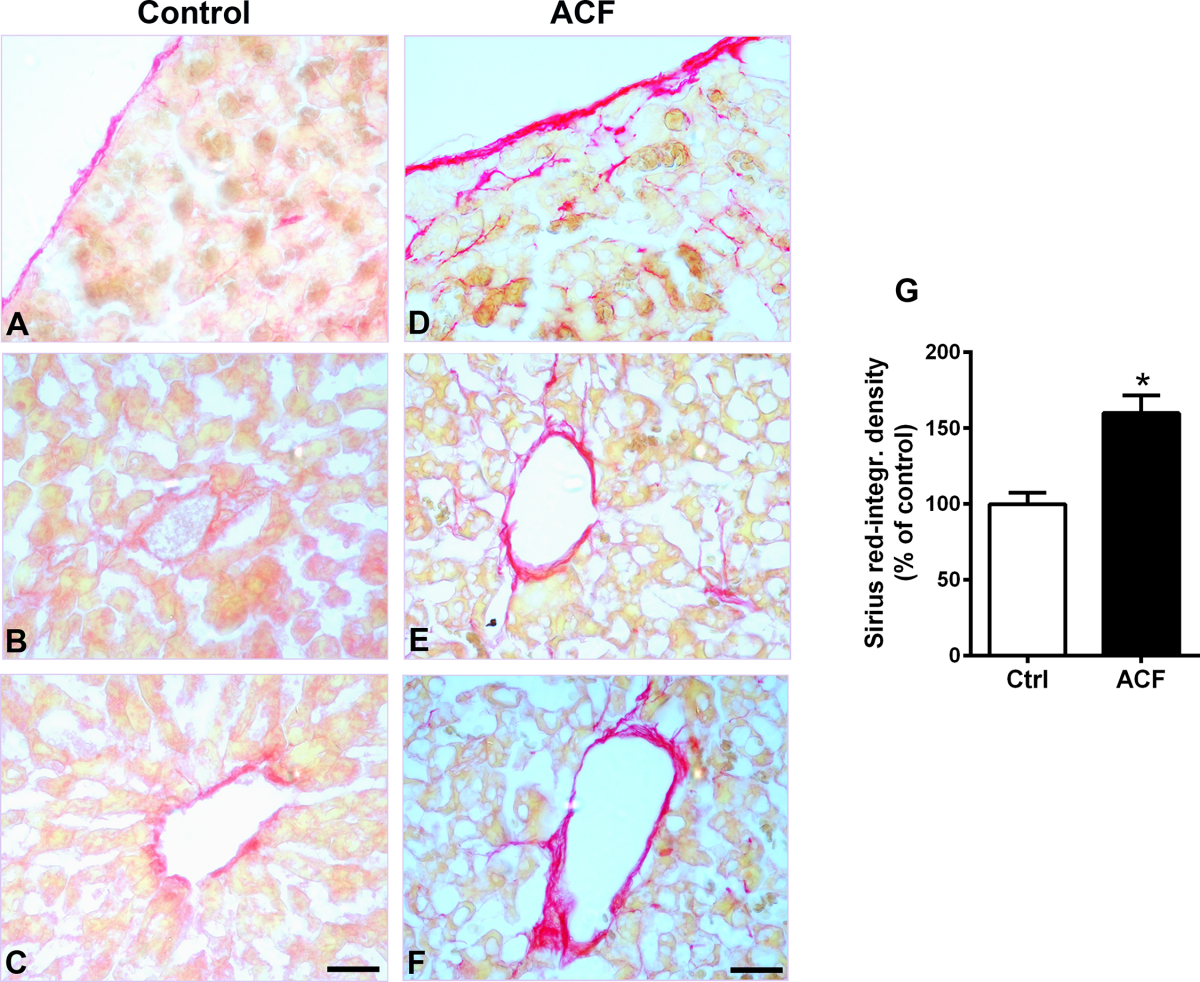
**Light microscopic photographs of representative Sirius red staining of liver sections in control (A-C) and ACF (D-F).** Note, representative Sirius red staining of liver sections shows extensive collagen deposition, indicating fibrosis progression in ACF rats (n = 5) compared to controls (n = 5) (**G**)(Sirius red-optical density ACF: 160±11.7% versus controls: 100±7.6%; p<0.05, Student t-test). Bars = 20 μm. Data show means ± SD.

Random fields of liver tissue sections of sham operated rats showed normal structure of hepatic lobules with a central vein, radiating cords of hepatocytes, prominent round nuclei, eosinophilic cytoplasm and normal slender Kupffer cells (Fig 2A). However, liver tissue sections of rats with ACF-induced heart failure exhibited histo-architectural distortion such as sinusoidal dilation with signs of congestion, irregular arrangement of hepatocytes within hepatic cords, hypertrophied Kupffer cells, hepatocellular destruction with obvious degeneration of nuclei and signs of apoptotic cell death (nuclear pyknosis or crescent-shaped condensation of nuclear chromatin). Some of the hepatic cells that made up the cords did not have regular borders and were fragmented (Fig 2A–2D). Liver semi-thin sections of control rats stained with toluidine blue showed silver parenchyma of normal aspect (Fig 2E). However, liver sections of ACF rats revealed more extensive areas of large vacuoles, heterogeneous liver parenchyma consisting of dark and compact hepatocytes flanked by degenerated cells, as well as hepatocytes with large vacuoles (Fig 2F). In addition, the cytoplasm of numerous hepatocytes with nuclear degeneration was pale or less stained with toluidine blue and showed more vacuolization (Fig 2F). These cells called ballooned cells or apoptotic cells and prove that the hepatocytes were undergoing an apoptosis process as described by [25]. In addition, there were leukocyte infiltrations within the sinusoids as detected by the pan-leukocyte marker CD45 (Fig 3A–3C). Also, glycogen granules of hepatic cells as examined in PAS-stained liver sections showed a marked decrease in glycogen granules in ACF rats compared to controls (Fig 3D–3F). In addition, Oil Red O stain highlighting fat globules in frozen sections of the liver revealed the presence of large (macrovesicular) globules only in liver sections of ACF rats compared to controls (Fig 3G–3I). Finally, collagen fibers within liver connective tissue examined by Sirius red staining showed signs of fibrosis in the perivascular as well as capsular area extending downwards into the liver parenchyma of ACF rats (Fig 4A–4G).

### Electron microscopic evidence for hepatocyte ultrastructural changes

**Fig 5:**
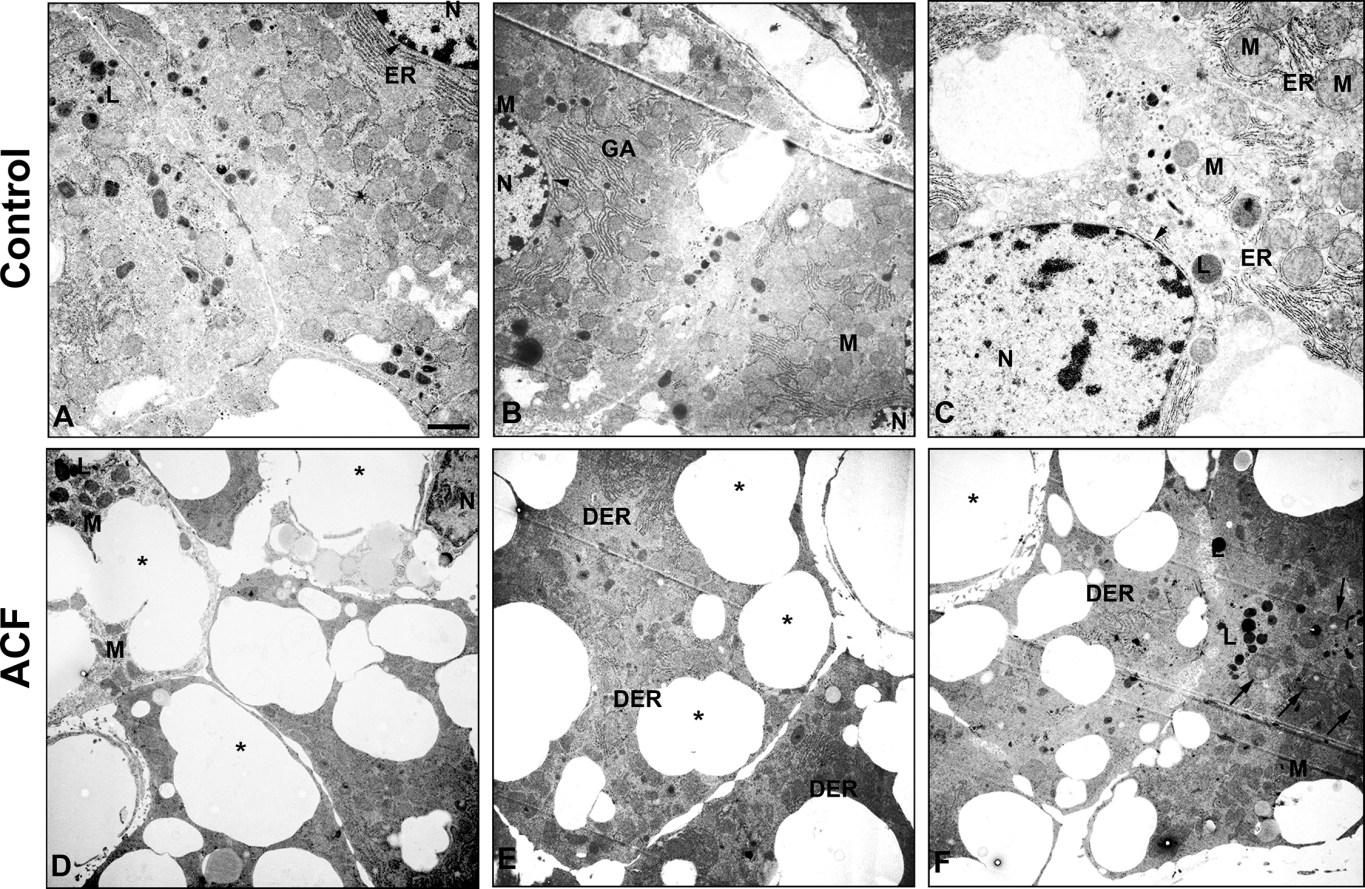
**Transmission electron micrograph of the rat liver in control (A-C) and ACF (D-F) rats. A-C)** Electron microscopic demonstration of control liver tissue. Liver cells of controls consist of round nuclei (N) with intact double-layered nuclear envelopes (arrowhead) (**A**), well-developed rough endoplasmic reticulum (ER) close to the nucleus, mitochondria (M), Golgi apparatus (GA), lysosomes (L) and a well-organized cytoplasm. **D-F**) Electron microscopic demonstration of ACF liver tissue shows cytoplasmic organelles are no longer distinguishable within hepatocytes. Ultrastructure evaluation shows marked cytoplasmic vacuolization with large vacuoles (*) of hepatocytes with irregular shaped nuclei (nucleus lost its rounded shape) (**D**), and degenerated fragmented rough endoplasmic reticulum (DER). **F**) Also, many shrunken mitochondria (M), became condensed (also known as apoptotic mitochondria) (arrow) in some hepatocytes. x5000; Bar:1 μm.

Ultrathin sections of livers from control rats stained with uranyl acetate and lead citrate present normal aspects of hepatocyte ultrastructure having prominent nuclei with intact double-layered nuclear envelopes, abundantly distributed healthy mitochondria, Golgi apparatus and widely distributed rough endoplasmic reticulum in close proximity to the nucleus (Fig 5A). In contrast, liver sections from ACF rats showed that cytoplasmic organelles were no longer distinguishable in many hepatocytes (Fig 5B–5F). Most of hepatocytes contained irregular shaped nuclei (lost their rounded shape), and degenerated fragmented rough endoplasmic reticulum. In addition, heavy vacuolization was visible in most of the hepatocellular cytoplasm (Fig 5B–5F). Also, many shrunken mitochondria became condensed (also known as apoptotic mitochondria) within hepatocytes (Fig 5B–5F).

### Evidence of apoptosis in liver cells following congestive heart failure

**Fig 6:**
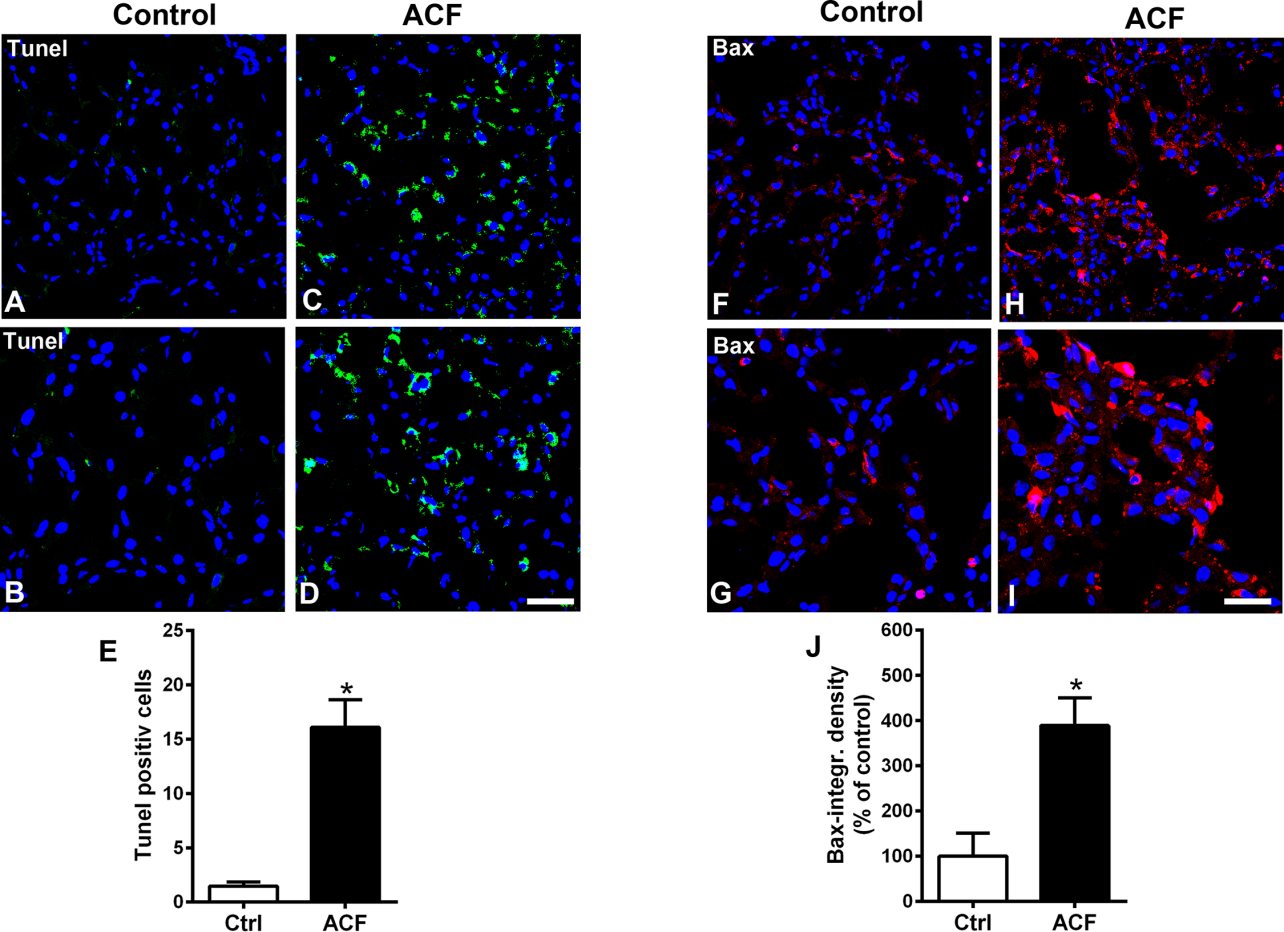
**Confocal microscopy of TUNEL staining (A-E) or proapoptotic Bax protein (F-J) in the liver of control and ACF rats. A-E**) showed TUNEL-positive (green fluorescence) with DAPI-counterstained nuclei (blue fluorescence) immunofluorescence of the liver in control or ACF adult rats. Note, apoptotic hepatic cells were detected in liver following ACF-induced heart failure in rats (**C and D**), however, no staining was found in controls (**A and B**)(Tunel positive cells per 200 μm^2^ ACF rats 16.1±2.5 versus Controls: 1.5±0.4; p<0.05, Student t-test). (**F-J**) Confocal microscopy of proapoptotic Bax protein (red fluorescence) with DAPI-counterstained nuclei (blue fluorescence) of the liver in controls or ACF rats. Note absent or weak Bax immunostaining in hepatocyte cytoplasm of controls (n = 5). In contrast in hepatocytes of ACF rats (n = 5), Bax immunofluorescence staining within hepatocyte cytoplasm is very prominent (Bax-optical density ACF rats 388.7±61.7 versus Controls 100±18%; p<0.05, Student t-test). Bars = 20 μm. Data show means ± SD.

Apoptosis was confirmed by histological TUNEL staining. In hepatic cells of control rats TUNEL staining was absent (Fig 6A and 6B). However, in hepatic cells of ACF rats, apoptosis was observed by intense TUNEL staining in condensed DAPI positive nuclei and significantly different compared to controls (p<0.05, Student t-test)(Fig 6C–6E).

### Expression of proapoptotic Bax and mitochondrial cytochrome C leakage into the cytosol of liver cells following congestive heart failure

**Fig 7:**
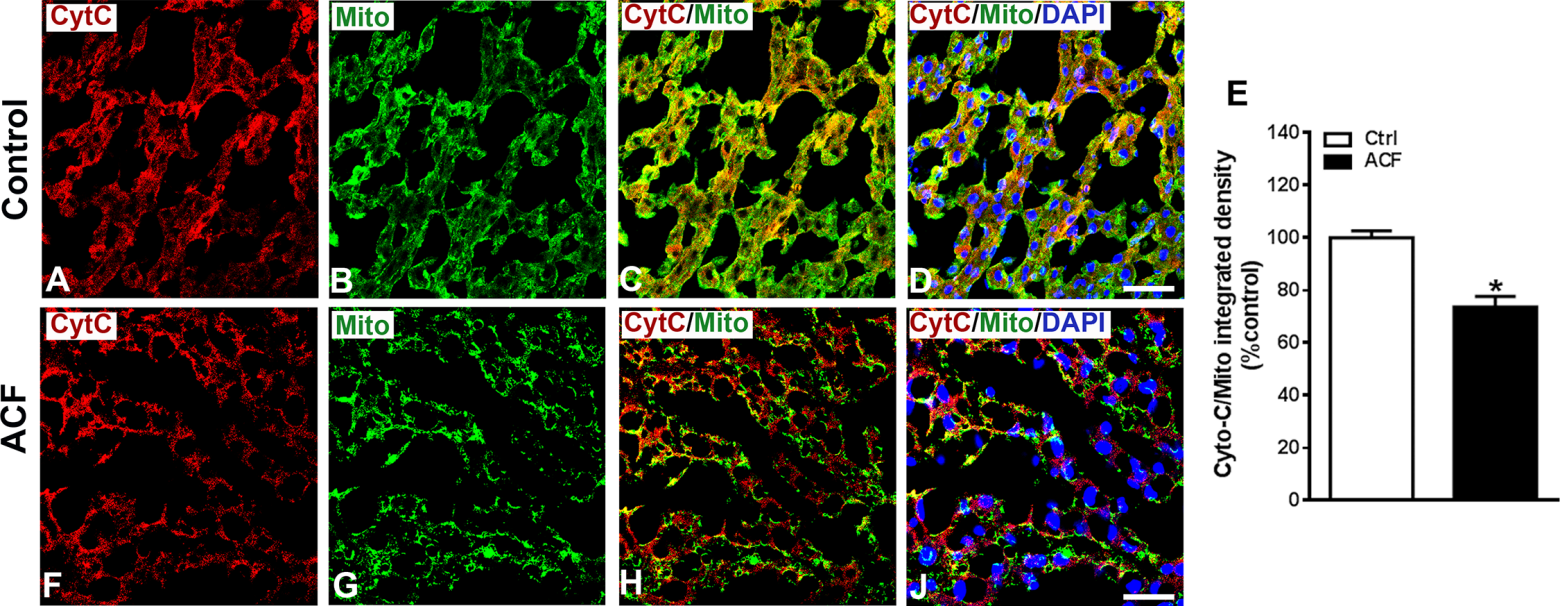
Confocal microscopy of cytochrome C (red fluorescence) with mitochondrial marker (green fluorescence) double immunofluorescence of liver sections of controls **(A-D)** or ACF **(E-H)** rats. Note that the cytochrome C immunostaining overlapped as indicated with yellow immunofluorescence) with the mitochondrial marker in the cytoplasm of hepatocytes of control animals **(C-D)**. However, in hepatocytes of ACF rats **(G-H)**, cytochrome C (red immunofluorescence) leaked from mitochondria and was distributed distinct from the mitochondrial marker (green immunofluorescence). **J)** Quantitative analysis of immunofluorescence microscopy of mitochondrial leakage of cytochrome C into the cytosol of liver cells following congestive heart failure. Note, the colocalization coefficient of cytochrome C and mitochondrial marker showed a significant reduction of their colocalization in the liver in ACF animals (n = 5) compared to controls (n = 5) (ACF rats 73.6±1.5 versus Controls: 100±1.9; p<0.05, Students *t*-test). Bars = 20 μm. Bars = 20 μm. Data show means ± SD.

Using immunofluorescence confocal microscopy, immunostaining of proapoptotic Bax protein was significantly increased in the hepatocytes of ACF rats (p<0.05, Student t-test)(Fig 6H and 6I). In hepatic cells of control rats, proapoptotic Bax protein was only faintly observed (Fig 6F and 6G). In the cytoplasm of hepatocytes of sham rats, cytochrome C immunoreactivity was predominantly restricted to mitochondria as revealed by an overlap with the mitochondrial marker using double immunofluorescence confocal microscopy (Fig 7A–7D). However, cytochrome C colocalization with the mitochondrial marker was only faint in liver sections of ACF animals and leaked into the surrounding cytosol (Fig 7F–7I). This apparent leakage of cytochrome C from mitochondria into the cytoplasm was evident in ACF rats, a process that is known to proceed the development of apoptotic cells death. Quantification of the colocalization coefficient of cytochrome C and mitochondrial marker showed a significant reduction of their colocalization in ACF animals compared to controls (p<0.01, Student t-test; control: 78±1.4 (100%); ACF: 57.5±1.4 (73.6%) (Fig 7E).

### Nuclear transfer of caspase 3 as activated caspase 3 into the nucleus of liver cells following congestive heart failure

**Fig 8:**
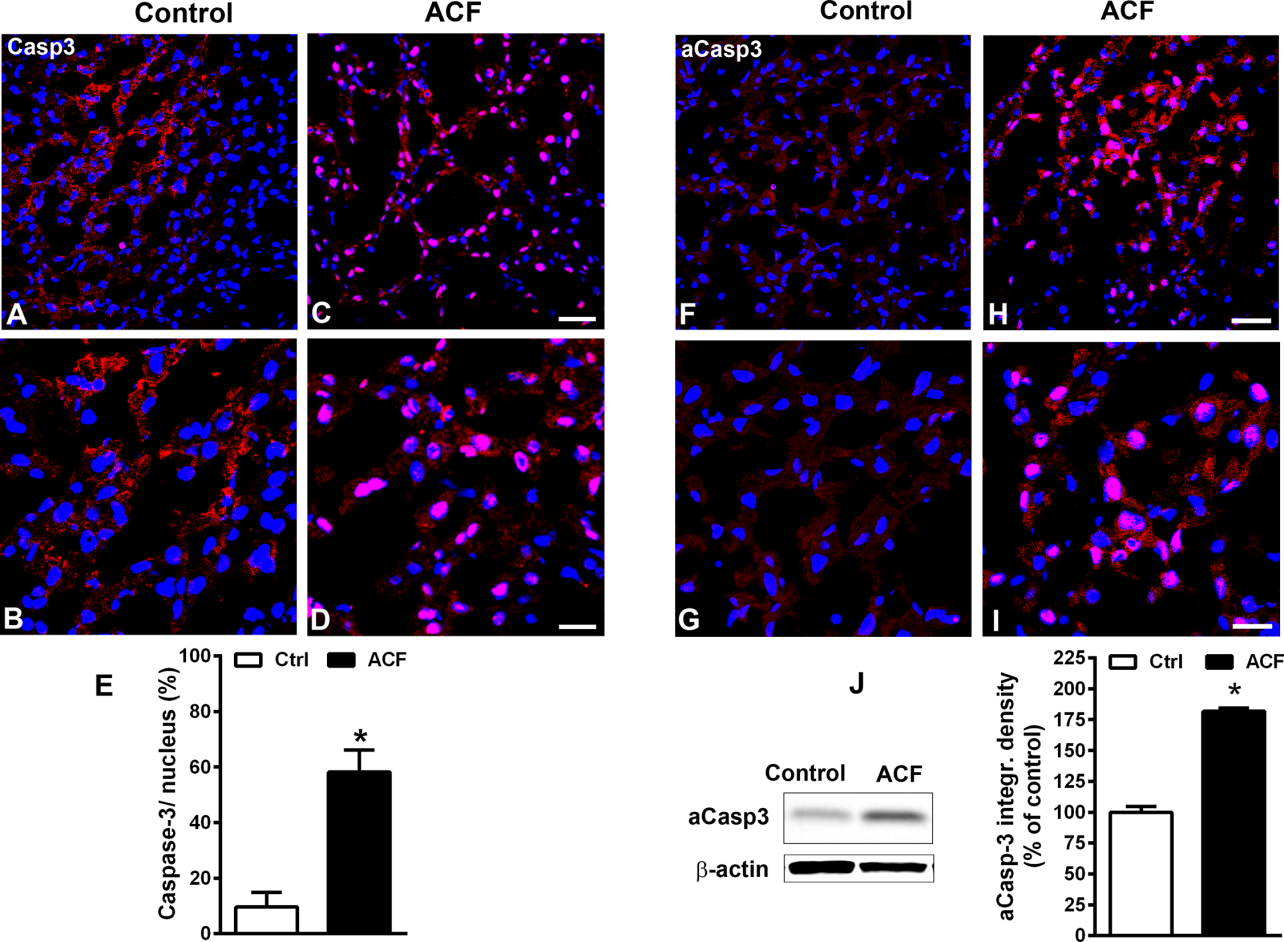
**Confocal immunofluorescence microscopy of caspase 3 (red fluorescence) using an antibody detecting pro-caspase 3 recombinant protein (A-D) or cleaved caspase 3 recombinant protein (F-I) with DAPI-counterstained nuclei (blue fluorescence) in liver sections of control or ACF adult rats.** Note, caspase 3 immunoreactivity was confined primarily to the well defined subcellular organelle-like structures in hepatic cells of control rats (**A, B**). In contrast in ACF rats, caspase 3 immunofluorescence was transferred to the perinuclear area of cells or inside nuclei of hepatic cells indicating an activation of pro-apoptotic factor caspase 3 (**C,D**). Confocal immunofluorescence microscopy activated caspase 3 (red fluorescence) and DAPI-counterstained nuclei (blue fluorescence) in liver sections using an antibody which detects exclusively cleaved caspase 3 recombinant protein. **H, I)** showed that cleaved caspase 3 immunoreactivity was confined primarily to the perinuclear area of cells or nuclei within hepatic cells of ACF rats, however, no staining was found in controls (**F, G**). Quantitative analysis of immunofluorescence microscopy of nuclear transfer of caspase 3 as activated caspase 3 into the nucleus of liver cells following congestive heart failure. Note, cleaved caspase 3-IR hepatocyte nuclei in the liver of ACF animals (n = 5) relative to controls (n = 5) showed a significant increase in the percentage of caspase 3-ir nuclei/total nuclei (ACF rats 58.2±2.8% versus Controls 9.6±1.8%; p<0.05, Students *t*-test)(**E**). Bar = 40 μm (**B, D, G, I**) and 20 μm (**A, C, F, H**). Western blot analysis for activated caspase3 protein with a molecular weight of 19 kDa in the liver of ACF and control rats. (**J**) The optical integrated density (OID) of activated caspase3 protein increased significantly in the liver of ACF rats (n = 5) compared to controls (n = 5) (ACF rats 181.8±2.7 versus Controls: 100±4.7%; p<0.05, Students *t*-test). Bars = 20 μm. Data show means ± SD.

Liver sections of control rats revealed that caspase 3 immunoreactivity was restricted primarily to well-defined subcellular organelle-like structures of hepatic cells (Fig 8A and 8B). In contrast in ACF rats, caspase 3 immunofluorescence was predominantly transfered to the immediate perinuclear area of cells or inside the nuclei of hepatic cells (Fig 8C–8E) indicating activation of the proapoptotic factor caspase 3. Therefore, we used the apoptotic marker cleaved activated caspase-3 which is detectable only during cell apoptosis [26]. Indeed, in using this antibody our Western blot analysis of respective liver tissue extracts showed a prominent single band of cleaved caspase 3 at the expected molecular weight of 19 kDa in ACF rats compared to a very faint or almost non-detectable band in controls (p<0.05, Student t-test)(Fig 8J) [19]. Immunohistochemical staining of liver sections of ACF rats revealed that cleaved caspase 3 immunoreactivity was confined primarily to the perinuclear area or within nuclei of hepatic cells (Fig 8H and 8I), however, no staining was found in control rats (p<0.05, Student t-test)(Fig 8F and 8G). Importantly, the number of caspase 3-IR nuclei as well as cleaved caspase 3-IR nuclei cells in relation to the total number of DAPI stained nuclei was significantly higher in ACF rats than controls (*p<0*.*05*, Student t-test) (Fig 8J).

## Discussion

Failure of the pumping heart often leads to the damage of other organs, especially the liver, and the combined dysfunction of heart and liver increases morbidity and mortality [1]. This study aimed to investigate possible mechanisms that underlie liver injury following heart dysfunction using the animal model of ACF-induced congestive heart failure [16, 19]. In this animal model heart failure developed in a predictable way within approximately 28 days. Animals revealed elevated central venous and left end diastolic pressures and increased rBNP-45 plasma concentrations indicating “backward failure” with pulmonary congestion [16, 19]. Liver tissue sections of ACF animals showed prominent hepatic damage including apparent congestion of blood vessels, increased breakdown of hepatocytes, cytoplasmic vacuolization concomitant with large lipid droplets and collagen fiber formation as well as glycogen depletion and hepatocyte ultrastructural alterations. Consistently, double immunofluorescent staining revealed a marked increase in the number of TUNEL-positive apoptotic hepatocytes concomitant with the enhanced expression of pro-apoptotic Bax, mitochondrial leakage of cytochrome C and nuclear transfer of activated caspase 3 indicating apoptotic processes and subsequent cell damage. These findings provide morphological evidence of apoptosis throughout damaged liver resulting from congestive heart failure.

Recently, our group modified an experimental rat model of heart failure to induce an infrarenal aortocaval fistula resulting in congestive heart failure within a predictable and short time period of 28 ± 2 days and without high mortality [16]. Consistent with our previous results [16], we found a significant increase in the heart and lung weight indices as well as rBNP-45 plasma concentrations in ACF animals compared to controls. In previous studies, the degree of heart and lung hypertrophy was directly correlated with hemodynamic changes of congestion including an increase in the left and right ventricular filling pressure as well as a significant reduction of left ventricular ejection fraction [16, 27]. Heart performance and liver function are closely interconnected and a synergistic relationship exists between these organs [3, 28]. Dysfunction of one organ often leads to a deterioration of function of the other [29]. During heart failure, chronic congestion is known to result in enhanced liver stiffness and subsequent liver fibrosis [4, 9]. In addition, the hemodynamic conditions may not be sufficient to achieve an adequate circulation of the liver, because inflow into the liver is limited by outflow pressure [30]. Therefore, congestive heart failure prevents the heart's ability to deliver the appropriate amount of oxygen to a rate proportionate to the demands of the metabolizing tissues that may result in damage to organs such as the liver [28, 31–34]. In a recent study, elevated serum levels of the peripheral cell death markers M65 and M30 gave first indirect evidence of hepatic apoptotic cell death in patients with liver injury following heart failure [1]. Here, we investigated the histopathological and ultrastructural changes of the liver as well as the expression of apoptotic cell death markers following heart failure.

We found that the histological liver structure in rats with ACF-induced heart failure exhibited apparent congestion of blood vessels, widespread apoptosis with obvious cytoplasmic vacuolization and degeneration of nuclei. Also, cytoplasmic glycogen granules were apparently decreased in ACF animals compared to controls. In contrast, collagen fibers in the connective tissue as well as large (macrovesicular) fat globules were apparently increased in ACF rats compared to controls. Besides liver damages observed with light microscopy, we also investigated the hepatocyte ultrastructural alterations. Indeed, the cytoplasmic organelles were no longer distinguishable in many hepatocytes of rats with congestive heart failure with obvious degenerated fragmented rough endoplasmic reticulum, shrunken mitochondria and heavy cytoplasm vacuolization. These findings are in agreement with a previous study using light microscopy which investigated the pathological changes in the liver obtained from patients with congestive heart failure [28]. The authors found in severe cases that liver cells showed a variety of degenerative changes including apoptosis and necrosis processes [28]. Also, these findings are consistent with the recent investigation by others [35, 3] which revealed a sinusoidal dilation and congestion with progressive fibrosis in liver biopsy from patients with heart failure.

TUNEL, i.e. terminal deoxynucleotidyl transferase-mediated dUTP nick end-labeling, has become a widely used staining method to assist in the detection of apoptotic cells of tissue sections. Indeed, our TUNEL apoptotic cell assay in liver tissue sections revealed a prominent increase in the number of TUNEL-positive apoptotic hepatocyte exclusively in ACF rats compared to controls. Apoptosis has long been proposed as a mechanism for hepatocellular damage in several liver disorders, including hepatitis, and alcoholic liver disease [36–38]. Therefore, the understanding of the cellular processes leading to programmed cell death (apoptosis) is of utmost importance in the study of liver disease. Recent evidence is emerging that the mitochondria-mediated apoptotic pathway is initiated by a variety of apoptosis-inducing signals to cause an imbalance of the major apoptosis regulators such as Bcl-2 and Bax [39]. Here, we provide first morphological evidence of the overexpression of the proapoptotic protein Bax in hepatocytes of rats following congestive heart failure. These findings are in agreement with the previous demonstration of Bax overexpression-accompanied apoptosis in response to liver injury induced by acetaminophen [40], liver cancer [41], psychoactive drug- induced hepatotoxicity [42], non-alcoholic rat liver disease [43] or chronic hepatitis [44].

Indeed, the previous studies reported that activation of the pro-apoptotic protein Bax triggers the disruption of mitochondrial transmembrane potential, followed by mitochondrial swelling and an increase in the permeability of the outer mitochondrial membrane [10, 11]. Consequently, the release of mitochondrial proteins like cytochrome C and the subsequent activation of caspase subtypes within the apoptosome complex activate downstream death programs [10, 11]. In line with this, our double immunofluorescence confocal microscopy revealed that the cytochrome C immunoreactivity was primarily confined to the mitochondria of control hepatocytes; however, its colocalization with the mitochondrial marker was clearly reduced in hepatocytes of ACF rats indicating leakage into the cytoplasm. These findings are in agreement with the previous study by Toledo [45] who found that oxidative stress is a common event in most hepatopathies leading to mitochondrial permeability transition pore formation and mitochondrial release of cytochrome C. This subsequently activates caspase 3 to finally result in hepatocytes death. Caspases (cysteinyl aspartate-specific proteases) are a family of important signaling molecules with various tasks depending on the subtype and organ involved [46]. Our recent study showed that the activation of caspases was a marker for cellular damage in diseases such as stroke and myocardial infarction [19]. Indeed, the appearance of the morphological and biochemical hallmarks of apoptosis and cell death [10] was associated with activation of caspase 3, i.e. the nuclear translocation of activated caspase 3 [11]. Therefore, we used the apoptotic marker cleaved activated caspase-3 which is absent under normal conditions and detectable only during cell apoptosis [26]. Indeed, cleaved caspase 3 was increased in ACF animals by Western blot as well as immunofluorescence microscopy, consistent with a previous demonstration of cleaved caspase 3 protein in rat heart [19]. Moreover, we demonstrated the translocation and, hence, activation of caspase 3 from cell organelle-like structures into the perinuclear area or nuclei of hepatocytes following heart failure induction.

In summary, taken together, the results demonstrate that ACF-induced congestive heart failure causes liver injury which results in sinusoidal dilation, breakdown of hepatocytes, cytoplasmic vacuolization concomitant with large lipid droplets and collagen fiber formation. In addition, hepatocellular apoptotic cell death mediated by the intrinsic pathway of mitochondrial cytochrome C leakage and subsequent transfer of activated caspase 3 into to the nucleus may occur to initiate overt DNA fragmentation and cell death. These findings provide evidence for progressive pathophysiological alterations of the liver during congestive heart failure.

## Supporting information

S1 Table The ARRIVE guidelines checklist. (PDF)

## References

[1] Herzer K, Kneiseler G, Bechmann LP, Post F, Schlattjan M, Sowa JP, et al Onset of heart failure determines the hepatic cell death pattern. Annals of hepatology. 2011;10(2):174–9. 21502679

[2] Fonarow GC, Adams KF Jr, Abraham WT, Yancy CW, Boscardin WJ. Risk stratification for in-hospital mortality in acutely decompensated heart failure: classification and regression tree analysis. Jama. 2005;293(5):572–80. doi: 10.1001/jama.293.5.572 15687312 10.1001/jama.293.5.572

[3] Horvath B, Zhu L, Allende D, Xie H, Guirguis J, Cruise M, et al Histology and Glutamine Synthetase Immunoreactivity in Liver Biopsies From Patients With Congestive Heart Failure. Gastroenterology research. 2017;10(3):182–9. doi: 10.14740/gr875e 28725306 10.14740/gr875e PMC5505284

[4] Mueller S. Does pressure cause liver cirrhosis? The sinusoidal pressure hypothesis. World journal of gastroenterology. 2016;22(48):10482–501. doi: 10.3748/wjg.v22.i48.10482 28082801 10.3748/wjg.v22.i48.10482 PMC5192260

[5] Allen LA, Matlock DD, Shetterly SM, Xu S, Levy WC, Portalupi LB, et al Use of Risk Models to Predict Death in the Next Year Among Individual Ambulatory Patients With Heart Failure. JAMA cardiology. 2016 doi: 10.1001/jamacardio.2016.5036 28002546 10.1001/jamacardio.2016.5036

[6] Gurwitz JH, Magid DJ, Smith DH, Goldberg RJ, McManus DD, Allen LA, et al Contemporary prevalence and correlates of incident heart failure with preserved ejection fraction. The American journal of medicine. 2013;126(5):393–400. doi: 10.1016/j.amjmed.2012.10.022 23499328 10.1016/j.amjmed.2012.10.022 PMC3627730

[7] Bechmann LP, Marquitan G, Jochum C, Saner F, Gerken G, Canbay A. Apoptosis versus necrosis rate as a predictor in acute liver failure following acetaminophen intoxication compared with acute-on-chronic liver failure. Liver international: official journal of the International Association for the Study of the Liver. 2008;28(5):713–6. doi: 10.1111/j.1478-3231.2007.01566.x 18433398 10.1111/j.1478-3231.2007.01566.x

[8] Canbay A, Jochum C, Bechmann LP, Festag S, Gieseler RK, Yuksel Z, et al Acute liver failure in a metropolitan area in Germany: a retrospective study (2002–2008). Zeitschrift fur Gastroenterologie. 2009;47(9):807–13. doi: 10.1055/s-0028-1109058 19750427 10.1055/s-0028-1109058

[9] Millonig G, Friedrich S, Adolf S, Fonouni H, Golriz M, Mehrabi A, et al Liver stiffness is directly influenced by central venous pressure. J Hepatol. 2010;52(2):206–10. doi: 10.1016/j.jhep.2009.11.018 20022130 10.1016/j.jhep.2009.11.018

[10] Budihardjo I, Oliver H, Lutter M, Luo X, Wang X. Biochemical pathways of caspase activation during apoptosis. Annual review of cell and developmental biology. 1999;15:269–90. doi: 10.1146/annurev.cellbio.15.1.269 10611963 10.1146/annurev.cellbio.15.1.269

[11] Deng J, Wang G, Huang Q, Yan Y, Li K, Tan W, et al Oxidative stress-induced leaky sarcoplasmic reticulum underlying acute heart failure in severe burn trauma. Free radical biology & medicine. 2008;44(3):375–85. doi: 10.1016/j.freeradbiomed.2007.09.023 17976387 10.1016/j.freeradbiomed.2007.09.023

[12] Goldstein JC, Waterhouse NJ, Juin P, Evan GI, Green DR. The coordinate release of cytochrome c during apoptosis is rapid, complete and kinetically invariant. Nature cell biology. 2000;2(3):156–62. doi: 10.1038/35004029 10707086 10.1038/35004029

[13] Ott M, Gogvadze V, Orrenius S, Zhivotovsky B. Mitochondria, oxidative stress and cell death. Apoptosis: an international journal on programmed cell death. 2007;12(5):913–22. doi: 10.1007/s10495-007-0756-2 17453160 10.1007/s10495-007-0756-2

[14] Seervi M, Joseph J, Sobhan PK, Bhavya BC, Santhoshkumar TR. Essential requirement of cytochrome c release for caspase activation by procaspase-activating compound defined by cellular models. Cell death & disease. 2011;2:e207 doi: 10.1038/cddis.2011.90 21900958 10.1038/cddis.2011.90 PMC3186908

[15] Garcia R, Diebold S. Simple, rapid, and effective method of producing aortocaval shunts in the rat. Cardiovascular research. 1990;24(5):430–2. 2142618 10.1093/cvr/24.5.430

[16] Treskatsch S, Feldheiser A, Rosin AT, Sifringer M, Habazettl H, Mousa SA, et al A modified approach to induce predictable congestive heart failure by volume overload in rats. PLoS One. 2014;9(1):e87531 doi: 10.1371/journal.pone.0087531 24498127 10.1371/journal.pone.0087531 PMC3909118

[17] Pacher P, Nagayama T, Mukhopadhyay P, Batkai S, Kass DA. Measurement of cardiac function using pressure-volume conductance catheter technique in mice and rats. Nature protocols. 2008;3(9):1422–34. doi: 10.1038/nprot.2008.138 18772869 10.1038/nprot.2008.138 PMC2597499

[18] Saha DC, Saha AC, Malik G, Astiz ME, Rackow EC. Comparison of cardiovascular effects of tiletamine-zolazepam, pentobarbital, and ketamine-xylazine in male rats. Journal of the American Association for Laboratory Animal Science: JAALAS. 2007;46(2):74–80. 17343357

[19] Treskatsch S, Shakibaei M, Feldheiser A, Shaqura M, Dehe L, Roepke TK, et al Ultrastructural changes associated with myocardial apoptosis, in failing rat hearts induced by volume overload. International journal of cardiology. 2015;197:327–32. doi: 10.1016/j.ijcard.2015.06.067 26159040 10.1016/j.ijcard.2015.06.067

[20] Mayer P. Ueber das Faerben mit Haematoxylin. Mitt Zool Stat Neapel. 1981;(10):170–86.

[21] Mousa SA, Shakibaei M, Sitte N, Schafer M, Stein C. Subcellular pathways of beta-endorphin synthesis, processing, and release from immunocytes in inflammatory pain. Endocrinology. 2004;145(3):1331–41. doi: 10.1210/en.2003-1287 14630714 10.1210/en.2003-1287

[22] Mousa SA, Shaqura M, Winkler J, Khalefa BI, Al-Madol MA, Shakibaei M, et al Protein kinase C-mediated mu-opioid receptor phosphorylation and desensitization in rats, and its prevention during early diabetes. Pain. 2016;157(4):910–21. doi: 10.1097/j.pain.0000000000000459 26713421 10.1097/j.pain.0000000000000459

[23] Shaqura M, Khalefa BI, Shakibaei M, Zollner C, Al-Khrasani M, Furst S, et al New insights into mechanisms of opioid inhibitory effects on capsaicin-induced TRPV1 activity during painful diabetic neuropathy. Neuropharmacology. 2014;85:142–50. doi: 10.1016/j.neuropharm.2014.05.026 24863039 10.1016/j.neuropharm.2014.05.026

[24] Manders EM, Verbeek FJ, Aten JA. Measurement of colocalization of objects in dual-color confocal images. J Microsc Oxford 1993;169:375–82. 10.1111/j.1365-2818.1993.tb03313.x 33930978

[25] Yip WW, Burt AD. Alcoholic liver disease. Seminars in diagnostic pathology. 2006;23(3–4):149–60. 17355088 10.1053/j.semdp.2006.11.002

[26] Yang M, Antoine DJ, Weemhoff JL, Jenkins RE, Farhood A, Park BK, et al Biomarkers distinguish apoptotic and necrotic cell death during hepatic ischemia/reperfusion injury in mice. Liver transplantation: official publication of the American Association for the Study of Liver Diseases and the International Liver Transplantation Society. 2014;20(11):1372–82. doi: 10.1002/lt.23958 25046819 10.1002/lt.23958 PMC4213307

[27] Tsunoda K, Hodsman GP, Sumithran E, Johnston CI. Atrial natriuretic peptide in chronic heart failure in the rat: a correlation with ventricular dysfunction. Circulation research. 1986;59(3):256–61. 2945670 10.1161/01.res.59.3.256

[28] Sherlock S. Cirrhosis of the liver. Postgraduate medical journal. 1950;26(299):472–83. 14776083 10.1136/pgmj.26.299.472 PMC2530301

[29] Donati A, Carsetti A, Damiani E. The role of cardiac dysfunction in multiorgan dysfunction. Current opinion in anaesthesiology. 2016;29(2):172–7. doi: 10.1097/ACO.0000000000000296 26705131 10.1097/ACO.0000000000000296

[30] Losser MR, Payen D. Mechanisms of liver damage. Seminars in liver disease. 1996;16(4):357–67. doi: 10.1055/s-2007-1007249 9027949 10.1055/s-2007-1007249

[31] Allen LA, Felker GM, Pocock S, McMurray JJ, Pfeffer MA, Swedberg K, et al Liver function abnormalities and outcome in patients with chronic heart failure: data from the Candesartan in Heart Failure: Assessment of Reduction in Mortality and Morbidity (CHARM) program. European journal of heart failure. 2009;11(2):170–7. doi: 10.1093/eurjhf/hfn031 19168515 10.1093/eurjhf/hfn031 PMC2639422

[32] Damman K, Navis G, Voors AA, Asselbergs FW, Smilde TD, Cleland JG, et al Worsening renal function and prognosis in heart failure: systematic review and meta-analysis. Journal of cardiac failure. 2007;13(8):599–608. doi: 10.1016/j.cardfail.2007.04.008 17923350 10.1016/j.cardfail.2007.04.008

[33] de Silva R, Nikitin NP, Witte KK, Rigby AS, Goode K, Bhandari S, et al Incidence of renal dysfunction over 6 months in patients with chronic heart failure due to left ventricular systolic dysfunction: contributing factors and relationship to prognosis. European heart journal. 2006;27(5):569–81. doi: 10.1093/eurheartj/ehi696 16364971 10.1093/eurheartj/ehi696

[34] McMurray JJ, Adamopoulos S, Anker SD, Auricchio A, Bohm M, Dickstein K, et al ESC guidelines for the diagnosis and treatment of acute and chronic heart failure 2012: The Task Force for the Diagnosis and Treatment of Acute and Chronic Heart Failure 2012 of the European Society of Cardiology. Developed in collaboration with the Heart Failure Association (HFA) of the ESC. European journal of heart failure. 2012;14(8):803–69. doi: 10.1093/eurjhf/hfs105 22828712 10.1093/eurjhf/hfs105

[35] Louie CY, Pham MX, Daugherty TJ, Kambham N, Higgins JP. The liver in heart failure: a biopsy and explant series of the histopathologic and laboratory findings with a particular focus on pre-cardiac transplant evaluation. Modern pathology: an official journal of the United States and Canadian Academy of Pathology, Inc. 2015;28(7):932–43. doi: 10.1038/modpathol.2015.40 25793895 10.1038/modpathol.2015.40

[36] Zhao X, Yang D, Peng Z. [Hepatocyte apoptosis in chronic viral hepatitis and its relationship with viral and Fas antigen expression]. Zhonghua nei ke za zhi. 1997;36(10):658–60. 10436978

[37] Benedetti A, Jezequel AM, Orlandi F. A quantitative evaluation of apoptotic bodies in rat liver. Liver. 1988;8(3):172–7. 3393066 10.1111/j.1600-0676.1988.tb00987.x

[38] Leist M, Gantner F, Bohlinger I, Tiegs G, Germann PG, Wendel A. Tumor necrosis factor-induced hepatocyte apoptosis precedes liver failure in experimental murine shock models. The American journal of pathology. 1995;146(5):1220–34. 7538266 PMC1869293

[39] Guo Y, Zhang W, Yan YY, Ma CG, Wang X, Wang C, et al Triterpenoid pristimerin induced HepG2 cells apoptosis through ROS-mediated mitochondrial dysfunction. Journal of BUON: official journal of the Balkan Union of Oncology. 2013;18(2):477–85. 23818365

[40] Fadda E, Yu CH, Pomes R. Electrostatic control of proton pumping in cytochrome c oxidase. Biochimica et biophysica acta. 2008;1777(3):277–84. doi: 10.1016/j.bbabio.2007.11.010 18177731 10.1016/j.bbabio.2007.11.010

[41] Liu Y, Chang CC, Marsh GM, Wu F. Population attributable risk of aflatoxin-related liver cancer: systematic review and meta-analysis. European journal of cancer (Oxford, England: 1990). 2012;48(14):2125–36. doi: 10.1016/j.ejca.2012.02.009 22405700 10.1016/j.ejca.2012.02.009 PMC3374897

[42] Behroozaghdam M, Hashemi M, Javadi G, Mahdian R, Soleimani M. Expression of bax and bcl2 Genes in MDMA-induced Hepatotoxicity on Rat Liver Using Quantitative Real-Time PCR Method through Triggering Programmed Cell Death. Iranian Red Crescent medical journal. 2015;17(11):e24609 doi: 10.5812/ircmj.24609 26732379 10.5812/ircmj.24609 PMC4698330

[43] Li CP, Li JH, He SY, Li P, Zhong XL. Roles of Fas/Fasl, Bcl-2/Bax, and Caspase-8 in rat nonalcoholic fatty liver disease pathogenesis. Genetics and molecular research: GMR. 2014;13(2):3991–9. doi: 10.4238/2014.May.23.10 24938610 10.4238/2014.May.23.10

[44] Tsamandas AC, Thomopoulos K, Zolota V, Kourelis T, Karatzas T, Ravazoula P, et al Potential role of bcl-2 and bax mRNA and protein expression in chronic hepatitis type B and C: a clinicopathologic study. Modern pathology: an official journal of the United States and Canadian Academy of Pathology, Inc. 2003;16(12):1273–88. doi: 10.1097/01.MP.0000097367.56816.5E 14681329 10.1097/01.MP.0000097367.56816.5E

[45] Toledo FD, Perez LM, Basiglio CL, Ochoa JE, Sanchez Pozzi EJ, Roma MG. The Ca(2)(+)-calmodulin-Ca(2)(+)/calmodulin-dependent protein kinase II signaling pathway is involved in oxidative stress-induced mitochondrial permeability transition and apoptosis in isolated rat hepatocytes. Archives of toxicology. 2014;88(9):1695–709. doi: 10.1007/s00204-014-1219-5 24614978 10.1007/s00204-014-1219-5

[46] Turk B, Stoka V. Protease signalling in cell death: caspases versus cysteine cathepsins. FEBS letters. 2007;581(15):2761–7. doi: 10.1016/j.febslet.2007.05.038 . 17544407 10.1016/j.febslet.2007.05.038

